# Highly Sensitive and Selective Room-Temperature H_2_S Detection Using N-Doped Carbon Nanofiber/Ni−ZnO Composites

**DOI:** 10.1021/acsaelm.5c01903

**Published:** 2025-12-01

**Authors:** Akshara Paras Parekh, Carlos Posada, Narayan Karmakar, Shilpa Jain, Guoliang Liu

**Affiliations:** † Department of Chemistry, 1757 Virginia Tech, Blacksburg, Virginia 24061, United States; ‡ 232024 Dwarkadas. J. Sanghvi College of Engineering, Mumbai 400056, India; § Department of Chemistry, 210758 Jai Hind College, Mumbai 400020, India; ∥ Macromolecules Innovation Institute, Virginia Tech, Blacksburg, Virginia 24061, United States; ⊥ Division of Nanoscience, Academy of Integrated Science, 1757 Virginia Tech, Blacksburg, Virginia 24061, United States

**Keywords:** electrospinning, nitrogen-doped carbon nanofibers, Ni−ZnO nanocomposites, room temperature sensing, H_2_S sensor, selectivity, stability

## Abstract

Nitrogen-doped carbon nanofibers (NCNF) possess nanostructures with high surface area and porosity, showing excellent sensing capabilities when additionally doped with semiconducting metal oxides. However, issues related to the processability of NCNF remain a challenge in developing gas-sensing electronics. Herein, we report the use of electrospinning of polymer precursors to fabricate NCNF impregnated with uniformly distributed Ni-ZnO nanoparticles. Structural and crystallographic analyses reveal synergistic effects of NCNF with Ni-ZnO, generating multiple active sites with enhanced gas absorptivity and catalytic activity. NCNF/Ni-ZnO nanocomposites detect various gases, showing high selectivity toward H_2_S at room temperature. NCNF with 15% Ni-ZnO doping show a rapid sensor response of 65.6% at 200 ppm with a response time of 9.2 s and a recovery time of 52.3 s. The sensor maintained stability, repeatability, and reduced humidity interference through a polynomial compensation strategy, achieving a deviation below 7%. These results highlight NCNF/Ni−ZnO hybrids as highly sensitive, selective, and stable nanomaterials for real-time H_2_S monitoring.

## Graphical abstract

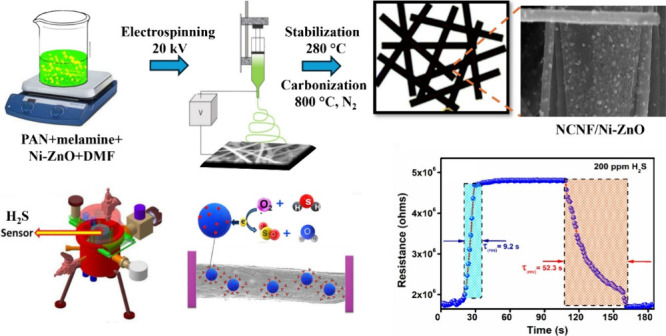

## 1 Introduction

The development of fast-responding sensors that can detect ppm and ppb levels of chemical substances is necessary in environmental monitoring, industrial safety, and medical diagnostics.^1,2^ Among them, hydrogen sulfide (H_2_S) is particularly hazardous. Exposure at levels as low as 10 ppm impairs cellular respiration, while concentrations beyond 25 ppm can cause severe neurological and respiratory damage.^3–6^ Although many commercial H_2_S sensors are available, they have several disadvantages, such as low sensitivity, short lifetimes, limited linear dynamic ranges, high power consumption, and poor selectivity.^7,8^ Therefore, developing room-temperature sensors with high sensitivity and selectivity is crucial for addressing these challenges. In recent years, considerable efforts have been made toward the development of semiconducting metal oxide (SMOs) based gas sensors for H_2_S detection, with notable materials such as WO_3_,^9^ ZnO,^10^ Fe_2_O_3_,^11^ In_2_O_3_,^12^ and SnO_2_.^13^ These materials leverage strategies such as noble metal doping, heterojunction formation, and nanostructuring, imparting good sensing behavior with tunable surface chemistry and strong redox reactivity. WO_3_-based systems have shown strong redox reactivity and tunable surface chemistry, and recent studies on oxygen-deficient WO_3_ demonstrate that engineered nonstoichiometry can activate localized surface plasmon resonance (LSPR) and significantly enhance charge-transfer kinetics for ultralow-level H_2_S detection.^14^ ZnO, in particular, exhibits high electron mobility, strong sensitivity to reducing gases, ease of nanostructuring, and chemical stability, enabling robust sensing behavior. However, pristine ZnO sensors typically operate at temperatures above 150 °C, leading to high power consumption along with poor humidity tolerance and limited selectivity.^15^ This poses safety risks in environments such as chemical plants, mines, and septic systems. Doping of active metals, such as Pd, Pt, Ag, Au, Cu, and Ni, introduces oxygen vacancies and catalytically active sites that significantly modulate charge transfer during gas adsorption.^16,17^ Similar defect-engineered behavior has been reported in ZnO systems, where the coupled formation of zinc interstitials (Zn_i_) and oxygen vacancies (V_O_) markedly enhances surface electron density and accelerates gas−solid charge transfer, resulting in significantly improved sensing performance.^18^ Yet, further strategies are required to simultaneously achieve room-temperature operation, fast response/recovery, and selectivity. One-dimensional (1D) carbon nanomaterials are fascinating due to their high aspect ratio, tunable conductivity, ability to operate at room temperature and strong gas−solid interactions under ambient conditions.^19–22^ Their porous, high-surface-area structure ensures uniform dispersion of nanoparticles, creating numerous active sites for gas interaction. This well-integrated architecture leads to efficient charge carrier mobility and strong gas−solid interactions. Nitrogen-doped carbon nanofibers (NCNF) significantly improve the surface reactivity, electrical and catalytic activity. This creates more active sites for gas adsorption than CNF.^23^ When coupled with SMOs, NCNF serve as conductive backbones, ensuring uniform nanoparticle dispersion and creating synergistic heterointerfaces that accelerate electron exchange with target gases. Such hybrid systems therefore combine the advantages of oxygen-vacancy engineering and conductive carbon frameworks, establishing a powerful design platform for next-generation sensors.

In this study, we report Ni-doped ZnO/NCNF nanocomposites as highly sensitive and selective room-temperature H_2_S sensors. Integration of Ni-ZnO with NCNF provides porous conductive networks and hydrophobic channels, mitigating humidity interference. When Ni ions substitute Zn^2^^+^ in the ZnO lattice, the slight ionic radius mismatch induces lattice distortion, which in turn increases the formation of oxygen vacancies to maintain charge neutrality. These oxygen-deficient sites act as shallow donor states, releasing free electrons into the conduction band and thereby increasing the electron concentration of the sensing layer. The elevated electron density improves the baseline conductivity and facilitates faster electron exchange with adsorbed gas molecules, resulting in higher response and shorter recovery times. Moreover, oxygen vacancies serve as preferential adsorption and reaction sites for oxygen species (O^−^, O^2−^) and target analytes, intensifying surface charge modulation during H_2_S exposure. Thus, nickel doping in ZnO introduces donor defects, enhancing charge separation, mobility, and gas sensitivity.^24–27^ In addition to the electronic effect, Ni species on the ZnO surface can catalyze the dissociation and oxidation of H_2_S molecules, further amplifying the sensing signal. These combined effects of oxygen vacancy enhanced charge carrier density, and catalytic activation have been widely reported in Ni−ZnO-based gas sensors^28,29^ and are consistent with our observed improvement in response for the 15% Ni−ZnO composition. The optimized NCNF/Ni−ZnO (15 wt %) composite exhibited a high response of 65.6% at 200 ppm of H_2_S, with a response time of 9.2 s and a recovery time of 52.3 s, outperforming pristine NCNF­(58s). Importantly, we demonstrate a mathematical humidity compensation approach, which reduces relative humidity induced deviations from ∼24 to ∼6%, ensuring reproducibility under fluctuating environments. Together, these findings establish NCNF/Ni−ZnO composites as energy-efficient and promising candidates for highly selective and efficient H_2_S detection.

## 2 Experimental Section

### 2.1 Materials and Synthesis of N-Doped Carbon Nanofiber/Ni-ZnO Nanocomposites

Analytical reagent (AR) grade chemicals, including zinc acetate (Zn­(CH_3_COO)_2_), nickel chloride (NiCl_2_), cetyltrimethylammonium bromide (CTAB), sodium hydroxide (NaOH), melamine, polyacrylonitrile (PAN), and ammonium persulfate (APS), were sourced from Merck Chemicals and used as received without additional purification.

The synthesis of Ni-ZnO was done using a wet chemical approach, as detailed in a previous report.^30^ To a solution of zinc acetate (25 mmol), 10 mL of CTAB solution was added under constant stirring for efficient capping of the Zn ions. Over 30 min, a NiCl_2_ solution was added dropwise while constant stirring. A NaOH solution (200 mmol) was slowly added, and the mixture was heated for ∼3 h at 80 °C. This process resulted in a colored solution, which was allowed to age for 1 day. The resulting precipitate was collected via centrifugation, washed thoroughly with deionized water and ethanol, and dried in a hot oven at 90 °C. For NCNF/Ni-ZnO, the synthesized Ni-ZnO was dispersed in 10 mL of DMF via ultrasound sonication for 30 min. After achieving complete dispersion, melamine (0.2 g) and PAN (1 g) were added to the homogenized mixture and stirred for 6 h at 60 °C to attain a proper viscosity for electrospinning. The polymer solution was loaded into a 10 mL syringe fitted with a 21-gauge needle. A voltage of 20 kV was applied between the syringe needle and a rotating drum (covered with aluminum foil) positioned 15 cm away from the needle tip. The polymer solution was fed at a constant rate of 1 mL/h during the spinning process. Nanofibers were collected on the drum and subsequently dried thoroughly at 60 °C in an oven to ensure complete evaporation of any residual solvent. The as-obtained fibers were stabilized by heating at 280 °C (ramp rate, 2 °C/min) for 3 h. Lastly, the preoxidized fibers were carbonized at 800 °C for 2 h under nitrogen flow to obtain N-doped carbon nanofibers/Ni-ZnO nanocomposites. To check the effect of Ni-ZnO on PAN nanofibers, different weight percentages of Ni-ZnO (5, 10, 15, and 20%) were added to the PAN solution. Nanofibers were synthesized using the same synthetic route, designated as NCNF/Ni-ZnO 5%, NCNF/Ni-ZnO 10%, NCNF/Ni-ZnO 15%, and NCNF/Ni-ZnO 20%, respectively. Similarly, NCNF was fabricated without adding any Ni-ZnO nanocomposite.

### 2.2 Structural Characterization

X-ray diffraction (XRD) measurements were performed to examine the crystalline structures of the nanofibers using a Rigaku Ultima IV powder diffractometer equipped with a Cu Kα source (λ = 1.5418 Å) and a 1.6 kW X-ray tube, operating at 40 kV and 40 mA. The crystalline peaks were matched to standard JCPDS data files. X-ray photoelectron spectroscopy (XPS) was performed on a PHI 5000 Versa Probe II to analyze the surface atomic composition. Monochromatic aluminum and an Al Kα X-ray source (*hv* = 1486.6 eV) were used to gather the XPS data in a 10^−7^ Pa vacuum. The 284.7 eV C 1s line was used to calibrate all observations of binding energy. Phi’s Multipak software was utilized to deconvolute the XPS peaks. The morphology and particle size were examined using a Hitachi S-4800 (Japan) field emission scanning electron microscope (FE-SEM). Transmission electron microscopy (TEM) was performed with a JEOL JEM 2100 to measure particle size. The Brunauer−Emmett−Teller (BET) method and nitrogen adsorption−desorption at 77 K were utilized to calculate the specific surface area. The results were recorded on an automated gas sorption analyzer (Autosorb-iQ).

### 2.3 Fabrication and Measurement of the Gas Sensor

For sensing measurements, two silver contacts (probes) with a 1 mm gap between the electrodes were employed. All measurements were performed in a static mode at ambient temperatures (∼26 °C) and varying relative humidities (RH) ranging from 10 to 90%. The sensor films were placed in a 1-L stainless steel chamber. With a gastight syringe, a measured volume of the designated gas was delivered to reach the necessary gas concentration (ppm). A Keithley Electrometer/High Resistance Meter Model 6517B was used to record variations in resistance over time during gas exposure to track the sensor’s response. By opening the lid of the static gas testing unit to the surrounding atmosphere, the samples were exposed to the ambient air, resulting in sensor recovery. Equation 1 was used to calculate the sensor response, where *R*_gas_ and *R*_air_ represent the resistances of the test gas and air, respectively.

$$\mathrm{Response}\;(\%)=\frac{R_{\mathrm{gas}}-R_{\mathrm{air}}}{R_{\mathrm{gas}}}\times 100$$ 1

The amount of time required for 95% of the total change in resistance upon exposure to gas and subsequent return to base resistance was used to establish response and recovery durations.^31,32^ A beaker containing a saturated solution, secured to the detecting dome, was used to maintain humidity. The saturated solutions of LiCl, MgCl_2_, Mg­(NO_3_)_2_, NaNO_2,_ and KCl were used to achieve humidity levels of 11, 32, 51, 63, and 84% RH, respectively.^33^

## 3 Results and Discussion

### 3.1 Structural, Morphological, and Compositional Analyses

Ni-ZnO-embedded NCNF nanocomposites were synthesized using an electrospinning process followed by carbonization under an N_2_ atmosphere (Figure 1a). A series of nanocomposites with varying Ni-ZnO concentrations (5, 10, 15, and 20%) relative to NCNF were prepared and labeled as NCNF/Ni-ZnO 5%, NCNF/Ni-ZnO 10%, NCNF/Ni-ZnO 15%, and NCNF/Ni-ZnO 20%, respectively.

**Figure 1:**
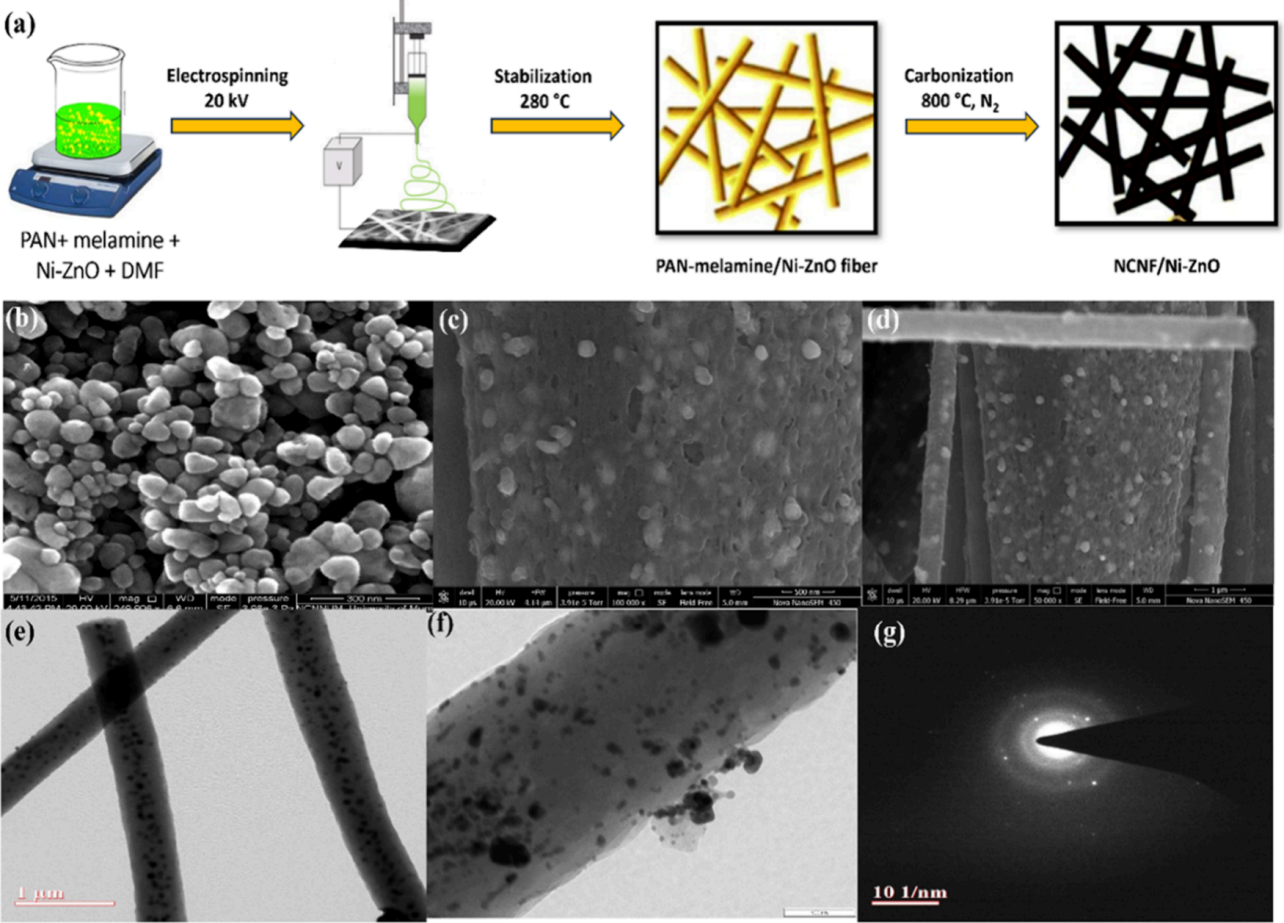
(a) Schematic overview of the fabrication process for NCNF/Ni-ZnO. (b) FE-SEM of Ni-ZnO. (c,d) SEM images, (e,f) TEM images, and (g) SAED of NCNF/Ni-ZnO 15%.

Scanning electron microscopy (SEM) images (Figure 1b) revealed that Ni-ZnO nanoparticles appeared as nanospheres with minimal aggregation. In NCNF/Ni-ZnO 15%, the Ni-doped ZnO spherical nanoparticles were dispersed across the surface of the carbon nanofibers, which had diameters ranging from 150 to 300 nm (Figure 1c,d). The Ni-ZnO nanoparticles display a degree of size and distribution heterogeneity, which is attributed to the electrospinning−calcination synthesis process. Although this variation may seem to compromise uniformity, it simultaneously generates numerous interfacial contact sites that promote improved gas adsorption and facilitate efficient electron transfer. Some particles were partially embedded within the fibers, indicating a strong interaction with the carbon matrix. The surface of the carbon nanofibers exhibited a rough texture, which may enhance the surface area and the number of active sites. Transmission electron microscopy (TEM) (Figure 1e,f) and selected-area electron diffraction (SAED) (Figure 1g) were employed to investigate the structural features of the nanocomposite fibers. TEM images showed a dense loading of spherical-like nanoparticles along the NCNF framework, with the nanoparticles anchored to the carbon structure. The SAED pattern showed no discernible lattice spacing, and the ring pattern confirmed the nanocomposite crystalline structure.

**Figure 2:**
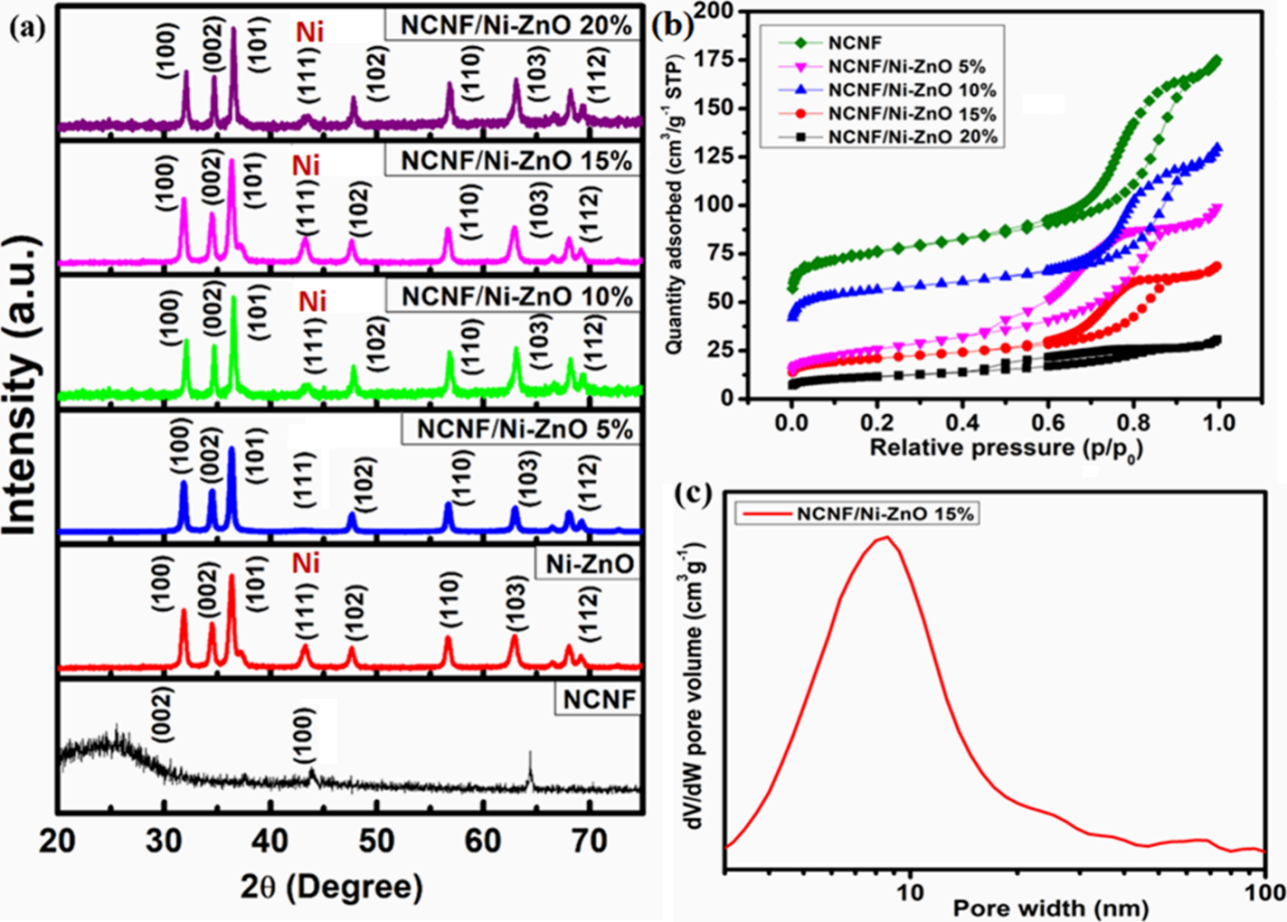
(a) XRD pattern, (b) N_2_ adsorption/desorption isotherms of NCNF/Ni-ZnO, in comparison with NCNF and Ni-ZnO. (c) Pore size distribution of NCNF/Ni-ZnO (15%).

Powdered X-ray diffraction (PXRD) was used to analyze the crystallographic nature of the nanocomposites. The XRD pattern of NCNF revealed a prominent reflection peak at 24.7° corresponding to the (002) plane of graphite, indicating the presence of graphite-like carbon. Additionally, a minor reflection peak at 44° associated with the (100) plane suggested an amorphous carbon component (Figure 2a). The XRD pattern of Ni-ZnO displayed distinct diffraction peaks at (100), (002), (101), (102), (110), (103), and (112) planes, corresponding to the hexagonal wurtzite structure of ZnO (JCPDS Card No. 36-1451).^34^ No peaks corresponding to metallic Zn were observed, confirming the absence of unreacted zinc. The peak at 44.5° (2θ) was attributed to Ni, indicating successful doping on the ZnO surface (JCPDS Card No. 03-1051).^35^ XRD measurements show the absence of any secondary phase diffraction peaks attributable to NiO, within the detection limits of the instrument. Furthermore, a slight shift in the (101) ZnO diffraction peak toward higher 2θ values is observed for Ni-doped samples, consistent with substitutional replacement of Zn^2^^+^ (ionic radius 0.74 Å) by the slightly smaller Ni^2^^+^ (0.69 Å) in the wurtzite ZnO lattice. The crystallite size of Ni-ZnO was calculated using the Debye−Scherrer formula, considering the three major diffraction peaks at (100), (002), and (101). The estimated average crystallite size was 31 nm. The XRD patterns for NCNF/Ni-ZnO composites with different Ni-ZnO ratios (5, 10, 15, and 20%) confirmed the successful integration of Ni-ZnO within the NCNF matrix, as evidenced by the diffraction peaks corresponding to Ni-ZnO and NCNF.

Brunauer−Emmett−Teller (BET) analysis further confirmed its porosity (Figure 2b). The isotherms displayed an H4 hysteresis loop, characteristic of type IV isotherms as per IUPAC classification, indicating the mesoporous nature of the nanofibers.^36^ The specific surface area of pure NCNF was measured to be 302 m^2^/g, which was higher than that of NCNF/Ni-ZnO composites with varying Ni-ZnO contents as 261 m^2^/g for NCNF/Ni-ZnO (5%), 243 m^2^/g for NCNF/Ni-ZnO (10%), 221 m^2^/g for NCNF/Ni-ZnO (15%), and 191 m^2^/g for NCNF/Ni-ZnO (20%). The reduced surface area is attributed to the incorporation of Ni-ZnO nanospheres, which partially block the pores of the NCNF matrix. According to the NLDFT pore-size distribution, NCNF/Ni-ZnO (15%) had mesopores in the range of 2−50 nm (Figure 2c). The mesoporous structure enhanced gas permeability and diffusion within the porous network, thereby increasing the availability of active sites for gas interaction. As a result, the NCNF/Ni-ZnO (15%) exhibited an improved gas-sensing performance due to its enhanced capacity for gas adsorption and diffusion.

**Figure 3:**
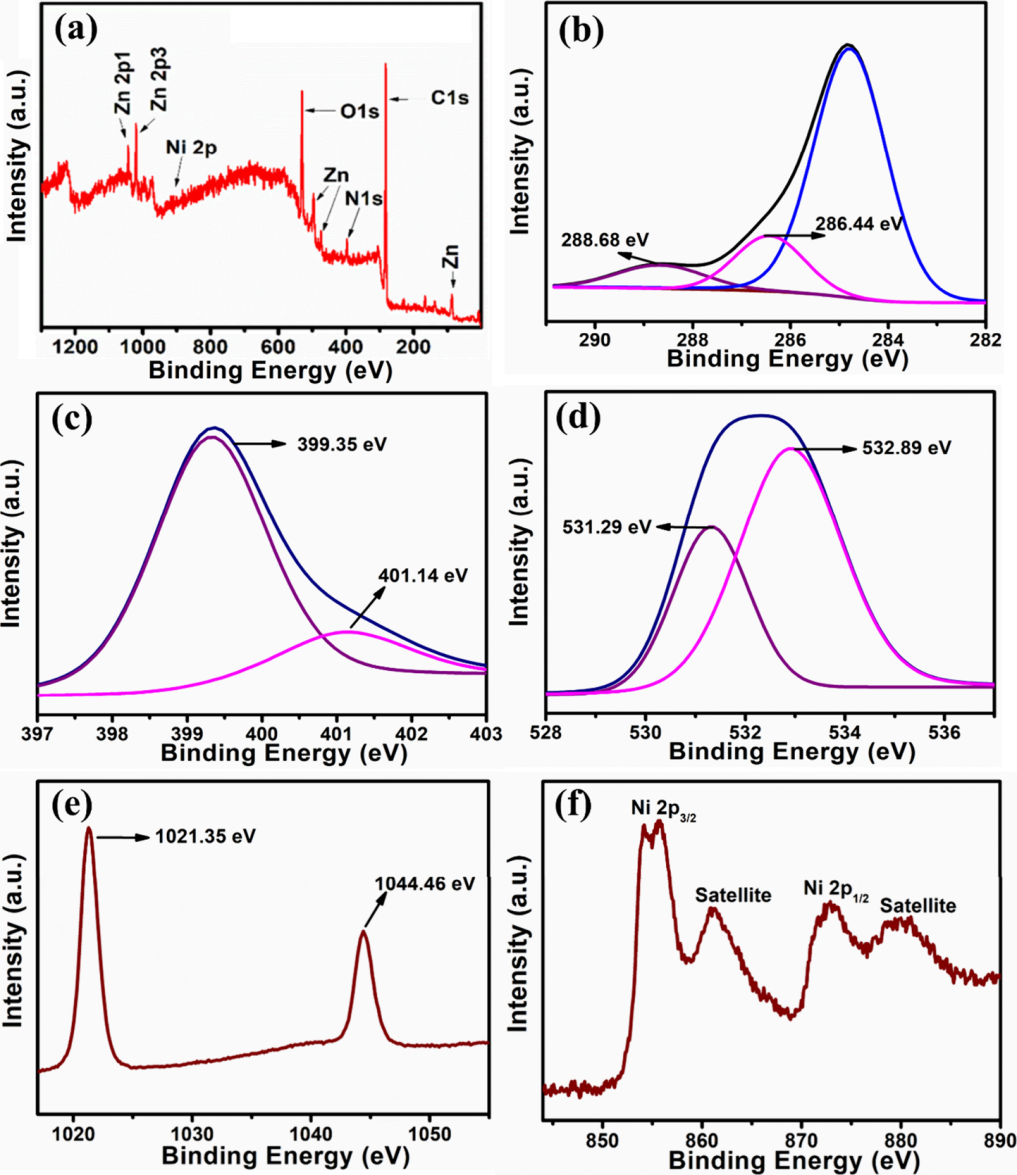
(a) XPS survey spectra of NCNF/Ni-ZnO (15%), (b) C 1s, (c) N 1s, (d) O 1s, (e) Zn 2p, and (f) Ni 2p.

X-ray photoelectron spectroscopy (XPS) was employed to investigate the surface chemical composition, providing insight into the degree of graphitization. The XPS survey (Figure 3a) revealed the presence of C 1s, O 1s, N 1s, Ni 2p, and Zn 2p orbitals in the NCNF/Ni-ZnO nanocomposite, where nitrogen and carbon originate from NCNF. Figure 3b shows the binding energies for NCNF/Ni-ZnO (15%), calibrated using C 1s (284.8 eV), along with the deconvoluted high-resolution C 1s spectrum. The deconvolution revealed a prominent peak at ∼286.44 eV associated with C−N, C−O, and C=O bonds.^37^ A peak at ∼288.68 eV was attributed to carboxyl groups (C−C=O).^38^ In the N 1s spectra, peaks at 399.35 and 401.14 eV corresponded to metal-N and C−N, respectively (Figure 3c). The O 1s peak at 532.89 eV corresponded to lattice oxygen (O^2−^) in ZnO, while a deconvoluted peak at 531.29 eV indicated oxygen vacancies in NCNF/Ni-ZnO (Figure 3d). Similar O 1s defect signatures have been reported in oxygen-deficient WO_3_ and ZnO enriched with Zn_i_ and V_O_, where higher O_II_ Oxygen Vacancies (V_o_) and O_III_ (Chemisorbed Oxygen Species) contributions indicate oxygen-deficient sites and chemisorbed oxygen associated with enhanced electron-donor defects.^14,18^ Other deconvoluted peaks were attributed to moisture adsorption, including O−H, Zn−O−H, and nonlattice oxygen species associated with point defects or oxygen vacancies.^39^ Zn 2p orbital showed two distinct peaks at 1021.35 eV (Zn 2p_3_/_2_) and 1044.46 eV (Zn 2p_1_/_2_), with a binding energy difference of 23.11 eV, confirming the presence of Zn^2^^+^ (Figure 3e). In the Ni 2p core spectrum, two prominent peaks at 872.88 eV (Ni 2p_1_/_2_) and 854.94 eV (Ni 2p_3_/_2_) were observed along with satellite peak associated with metallic Ni.^40^ The observed satellite peak is attributed to charge-transfer transitions, specifically from O 2p to Ni 3d orbitals.^41^ (Figure 3f). These positions and spectral shapes are characteristic of Ni ion species incorporated into the ZnO lattice,^42^ suggesting complete reduction during pyrolysis in an inert atmosphere. Ni^0^ enhances H_2_S sensing by catalyzing gas dissociation and improving electron transfer through conductive bridging between ZnO and NCNF. Here, XRD, XPS, and BET analyses collectively provide a comprehensive understanding of the crystallinity, surface chemistry, doping levels, and mesoporous structure of the nanocomposites, which are crucial for optimizing their performance in gas sensing.

### 3.2 Sensing Studies

The adsorption of gas molecules onto NCNF surfaces alters their electrical properties, resulting in measurable changes in conductivity or resistance.^20,21^ In addition, the tunable nitrogen doping in NCNF and integration with Ni-ZnO lead to a sensing material with improved selectivity, rapid response times, and low detection limits.^43,44^ With this rationale, after synthesizing NCNF with different ratios of Ni-ZnO (5, 10, 15, and 20% by weight), we evaluated various gas-sensing parameters, including selectivity, sensitivity, humidity, concentration, and stability. Initially, a selectivity study was conducted using different oxidizing and reducing gases and solvents, such as ammonia, nitrogen dioxide, acetone, methanol, and ethanol at 200 ppm and 32% RH. NCNF/Ni-ZnO (15%) shows the highest selectivity for H_2_S gas over other gases, with a maximum response of 63% (Figure 4).

**Figure 4:**
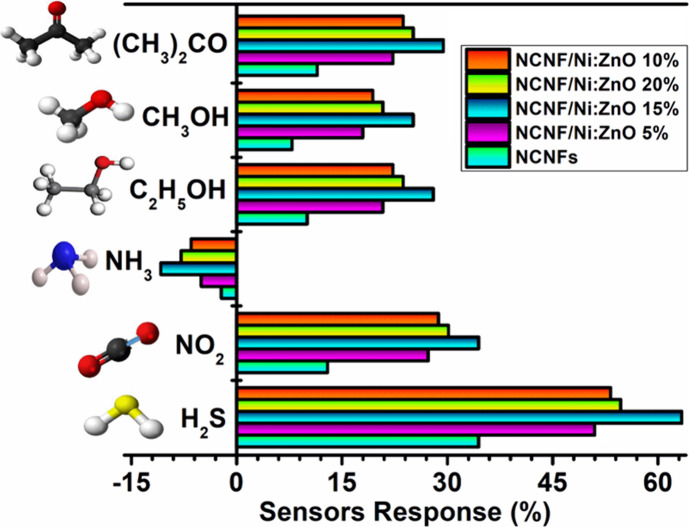
Selectivity of NCNF and NCNF/Ni-ZnO (5−20%) toward 200 ppm of different gases.

All composites exhibited high sensitivity to H_2_S gas in the concentration range of 50−500 ppm, and they consistently detected H_2_S levels as low as 50 ppm with a response of ∼10% (Figure 5c). NCNF showed a 51% response to 200 ppm of H_2_S, with a response time of 58 s and a recovery time of 84 s (Figure 5c). With 5% doping of Ni-ZnO in NCNF, sensor response increased from 51.0 to 56.7%. Further increasing Ni-ZnO doping to 10% yielded a response of 61.1%. Notably, NCNF/Ni-ZnO(15%) showed the highest response, and the sensor response increased with gas concentration: 65.6, 75.4, and 77.3% response at 200, 400, and 500 ppm, respectively (Figure 5a). The response time was 9.2 s, and the recovery time was 52.3 s (Figure 5b). Further increasing the doping to 20%, the sensor response decreased to 62.2% at 200 ppm of H_2_S gas. In Figure 5c, the sensing response varies with the Ni-ZnO loading, where 15 wt % exhibits optimal performance. This trend highlights the importance of balancing particle dispersion and active site accessibility. Higher loadings may lead to aggregation and hinder gas diffusion, while lower loadings may not provide a sufficient catalytic surface. Thus, the morphology observed plays a critical role in performance optimization. Despite these variances, the 15 wt % Ni-ZnO composite showed peak performance, suggesting that an optimal balance between Ni loading and dispersion facilitates faster response and recovery times. Higher doping levels increased charge carrier scattering, impairing the material’s electrical properties and overall sensor performance.^45^

There was a significant decrease in response time and recovery time with Ni-ZnO doping in the NCNF matrix. The response time was 58 s for NCNF, which decreased to 38.4 s for 5% doping, 23.7 s for 10% doping, and 9.2 s for 15% doping of Ni-ZnO. However, the response time increased to 16.5 s upon further increasing the doping concentration to 20%. Figure 5d presents a comparative analysis of NCNF and NCNF/Ni-ZnO with varying Ni-ZnO concentrations. While all NCNF/Ni-ZnO composites showed better response and faster recovery than pure NCNF, the optimal doping concentration appeared to be 15%, providing the best balance of sensitivity, stability, and response time.

**Figure 5:**
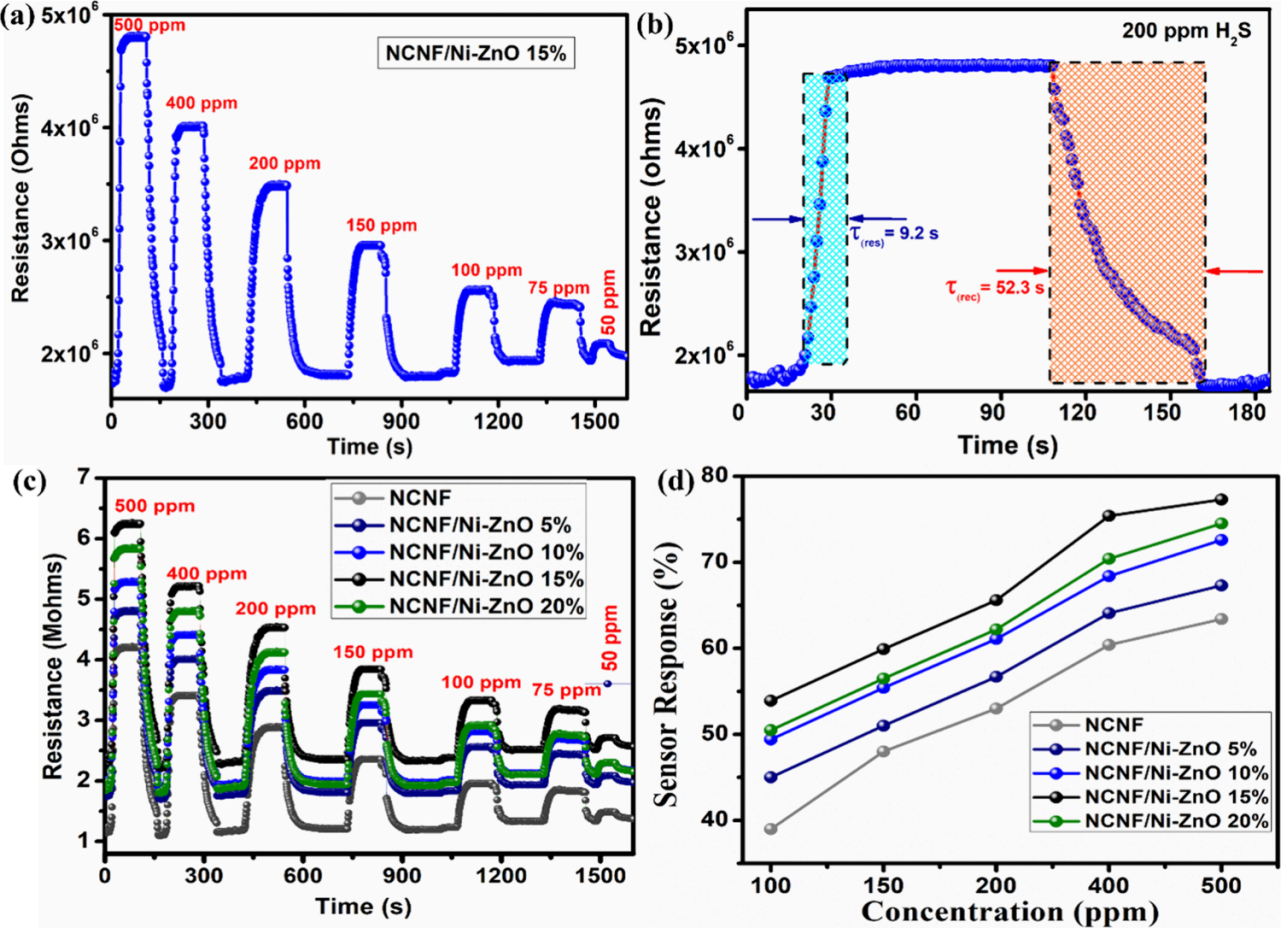
(a) Dynamic transient resistance curves of NCNF/Ni-ZnO (15%). (b) Response and recovery curves of NCNF/Ni-ZnO (15%) at 200 ppm of H_2_S. (c) Dynamic transient resistance curves of NCNF, NCNF/Ni-ZnO (5, 10, 15, 20%) toward H_2_S at different concentration. (d) Comparative sensor response of various nanocomposites.

The gas sensor performance under varying relative humidity (RH) is a critical parameter for reliable deployment in real-world environments. The impact of humidity on the gas-sensing response was studied (Figure 6a) in the range of RH 11−84% at room temperature, using saturated salt solutions of LiCl, MgCl_2_, Mg­(NO_3_)_2_, NaNO_2,_ and KCl, respectively. Before testing the conditions of elevated humidity, each solution was contained in a 5-L chamber and sealed for over 24 h.^46^ For NCNF/Ni−ZnO(15%), the baseline resistance was observed to decrease progressively with increasing RH, a trend attributed to the proton hopping mechanism facilitated by adsorbed water layers. When RH exceeded 50%, the sensor surface became increasingly coated with water molecules. Under an applied electric field, these molecules ionize, producing hydroxyl ions (OH^−^) and hydronium ions (H_3_O^+^) according to eqs 2 and 3.

$$2H_{2}O\to H_{3}O^{+}+{\mathrm{OH}}^{-}$$ 2

$$H_{3}O^{+}\to H_{2}O+H^{+}$$ 3

The initial hydronium ions migrate along the surface of carbon nanofibers and undergo further hydrolysis to form H_3_O^+^ (eqs 2 and 3), which subsequently releases mobile protons (H^+^) with high diffusion coefficients. These protons hop between neighboring adsorption sites, enabling rapid charge transport within the liquid-like conduction channels formed by adsorbed water molecules (Figure 6c). At low RH levels, the limited water coverage suppresses proton hopping, resulting in stable baseline resistance and minimal RH influence. However, at high RH, the increased protonic conductivity contributes to reduced sensor resistance. The NCNF/Ni−ZnO (15%) composite exhibited a baseline response of 77% at 11% RH, which decreased monotonically to 58% at 84% RH, corresponding to a deviation of 24.7%. This decrease is attributed to competitive adsorption between H_2_O molecules and H_2_S on active surface sites, resulting in a reduction in the surface concentration of chemisorbed oxygen species and, consequently, a lower modulation of the depletion layer. To quantitatively correct RH-induced drift, the measured response values at various RH were fitted to a second-order polynomial­(SI). This compensation reduced the deviation across 11−84% RH from 24.7 to 6.2%, with all corrected values falling within 76−78% (Table S1). The coefficient of variation (CV) value for the uncompensated sensor was 12.05%, which is competitive with recent H_2_S sensors incorporating Cu_2_O/ZnO heterostructures or CNT−CuO−ZnO films under similar RH conditions.^47,48^ Mechanistically, the residual RH dependence is likely due to physisorption of water on ZnO, which alters the surface dipole layer and partially blocks oxygen adsorption sites. The NCNF network contributes to RH resilience by providing hydrophobic pathways for gas diffusion and limiting direct water adsorption on active ZnO−Ni interfaces. We quantified humidity effects by measuring the H_2_S response at 200 ppm across relative humidity levels of 11−84% and applying a second-order polynomial compensation calibrated to the 11% RH baseline. As expected from proton hopping on adsorbed water layers, the raw sensor response decreased monotonically with increasing humidity. To quantitatively correct this drift, the measured values were fitted using a least-squares second-order polynomial of the form (eq 4):

$$R_{\mathrm{pred}}(\mathrm{RH})=a(\mathrm{RH}{)}^{2}+b(\mathrm{RH})+c$$ 4

where *R*_pred_(RH) is the predicted response at a given RH (%). The fitting yielded an excellent correlation (*R* ^2^ = 0.992; Table S1), validating the polynomial model. Compensation using the derived correction factor effectively flattened the humidity-induced response drift and enabled direct comparison of intrinsic sensor performance. For the NCNF/Ni−ZnO (15 wt %) composite, the raw response dropped from 77% at 11% RH to 58% at 84% RH, whereas the compensated values reduced the deviation from 24.7 to ∼6% (residual ∼ ±3%), with the coefficient of variation improving from 12.05 to 2.6%. These results confirm that the polynomial compensation approach efficiently suppresses humidity-related signal fluctuations, ensuring reliable real-world sensing performance under varying atmospheric moisture.

Figure 6b illustrates the stability of NCNF/Ni-ZnO(15%) when exposed to 500 ppm of H_2_S for 30 days, showing a gradual decrease with an average response of 67.6%. This sustained performance can be attributed to the enduring stability of carbon nanomaterials. The limit of detection (LOD) for H_2_S was calculated using the formula LOD = 3σ/slope, where σ is the baseline standard deviation and *S* is the slope of the response curve. Based on this calculation, the LOD for H_2_S was determined to be approximately 43 ppm, while the limit of quantification (LOQ), calculated as 10σ/slope, was approximately 131 ppm. These results confirm that the NCNF/Ni-ZnO sensor has the potential for detecting low concentrations of H_2_S in ambient environments. To assess operational robustness under fluctuating ambient humidity, NCNF/Ni−ZnO (15%) sensors were subjected to three consecutive RH variation cycles between 11 and 84% while exposed to 200 ppm of H_2_S (Figure S2). The response trend in each cycle closely overlapped, with minimal cycle-to-cycle drift (<3% variation), confirming the reproducibility of sensor behavior under dynamic humidity conditions. This stability under RH fluctuations further supports the humidity-resilient characteristics and demonstrates the suitability of the proposed RH compensation method for real-world environments.

**Figure 6:**
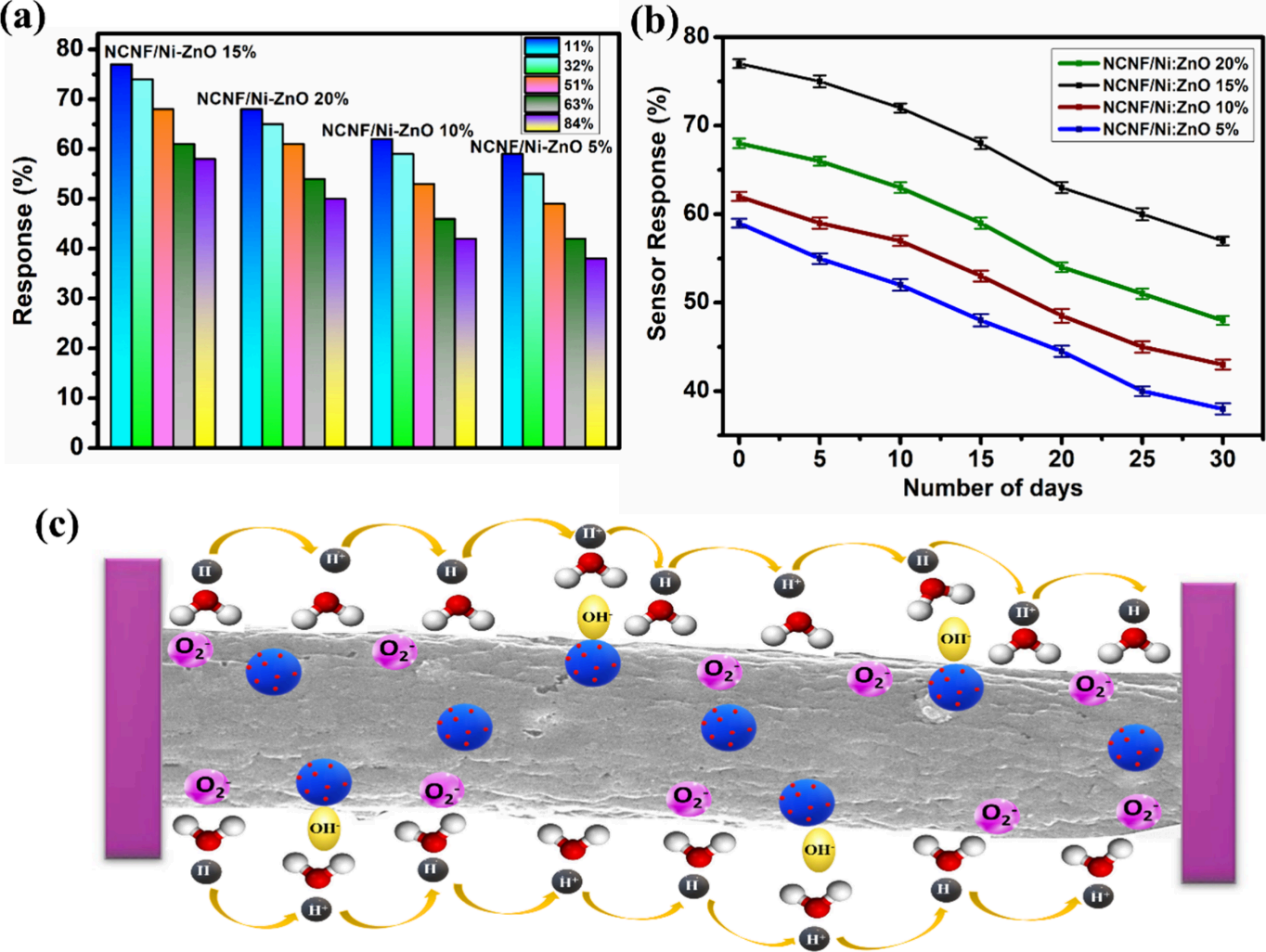
(a) Sensing response of NCNF/Ni-ZnO (5, 10, 15, 20%) toward H_2_S at 200 ppm with varying humidity levels. (b) Stability of NCNF/Ni-ZnO (5, 10, 15, 20%) toward 500 ppm of H_2_S. (c) Schematic representation of the proton hopping mechanism at high humidity levels.

### 3.3 Sensing Mechanism

The reactions governing the adsorption of oxygen on the nanocomposite surface and its interaction with H_2_S are outlined below (eqs 5–9):

$$O_{2(\mathrm{gas})}+e^{-}↔O_{2(\mathrm{chemisorbed})}^{-}$$ 5

$$O_{2(\mathrm{gas})}+2e^{-}↔2O_{\left(\mathrm{chemisorbed}\right)}^{-}$$ 6

$$O_{2(\mathrm{chemisorbed})}+2e^{-}↔O_{2(\mathrm{chemisorbed})}^{2-}$$ 7

$${O^{-}}_{\left(\mathrm{chemisorbed}\right)}+e^{-}↔O_{\left(\mathrm{chemisorbed}\right)}^{2-}$$ 8

$$2H_{2}S_{\left(\mathrm{chemisorbed}\right)}+3O_{2}^{-}↔2H_{2}O+2{\mathrm{SO}}_{2}+3e^{-}$$ 9

The work functions of n-type ZnO (∼5.2 eV)^49^ and p-type carbon nanofibers (CNF, ∼4.6 eV)^50^ support the formation of a nanoscale p−n heterojunction within the NCNF/Ni-ZnO composite. Doping ZnO with nickel introduces donor-type defects, such as oxygen vacancies and zinc interstitials, that enhance the carrier concentration in the conduction band by acting as electron donors. These defect sites improve the adsorption of both oxygen molecules and target analytes, such as H_2_S. Additionally, the interface between Ni and ZnO creates a p−n junction that gives rise to a Schottky barrier, regulating the depletion layer and enhancing charge separation when exposed to gas molecules. Ni domains also function as catalytic sites for H_2_S decomposition, facilitating a spillover mechanism whereby dissociated fragments migrate to adjacent ZnO surfaces for further reaction. This cooperative interaction significantly enhances gas sensing performance at ambient temperatures. The difference in Fermi levels between doped ZnO and NCNF drives electron flow at their interface. At room temperature, doped ZnO undergoes predominant ionization due to interstitial zinc atom pairs. When exposed to air, oxygen molecules adsorb onto the surface of Ni-ZnO nanoparticles deposited on NCNF. These oxygen molecules capture electrons, transforming into O_2_^−^ ions, as illustrated in Figure 7a,b. This electron depletion causes the space charge region of the heterojunction to widen, as shown in Figure 7c,d, further constricting the electron channel and increasing resistance. The NCNF/Ni-ZnO nanocomposite features numerous active sites, such as vacancies and structural defects, which enhance its gas adsorption capacity.^51^ These sites facilitate the adsorption of H_2_S gas and promote interactions with oxygen ions, accelerating the sensor’s response. This interaction reduces the resistance as electrons are released into the conduction band. The interconnected NCNF structure supports charge transport between heterojunctions, ensuring stable operation. The sensor’s recovery time is influenced by the concentration of H_2_S gas. Higher concentrations of H_2_S prolong the desorption process due to the slower desorption coefficients of gas molecules. However, the NCNF/Ni-ZnO sensor demonstrates a faster response time than the NCNF sensor alone. Various bimetallic nanocomposite flexible gas sensors for H_2_S detection in the works of the past years have been summarized in Table 1. It reveals that the NCNF/Ni-ZnO flexible gas sensor shows the merits of high response, fast response−recovery times, and ultralow detection limit for H_2_S monitoring at room temperature.

**Figure 7:**
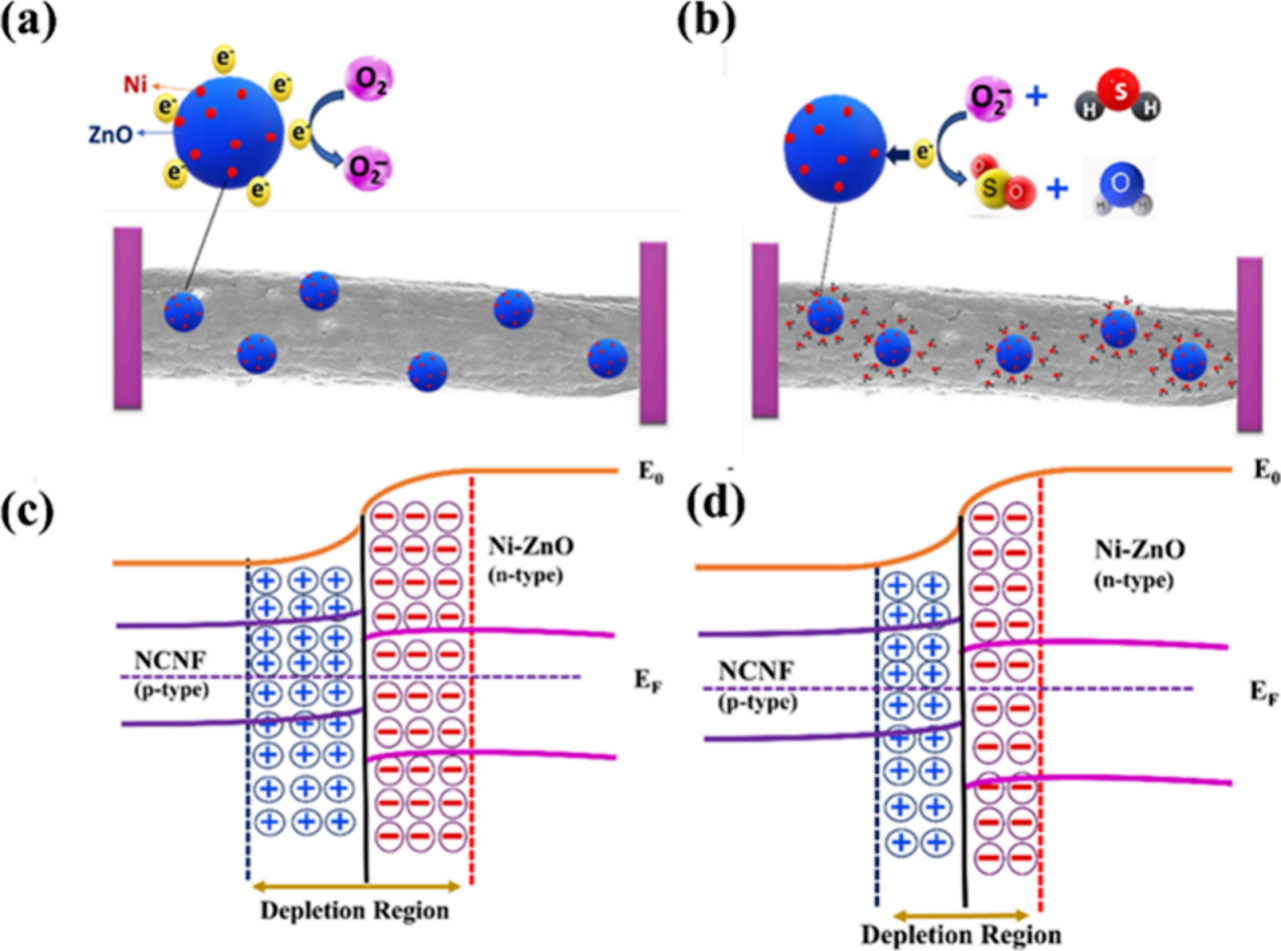
Schematic representation of the sensing mechanism of NCNF/Ni-ZnO (15%) in (a) air and (b) H_2_S. The energy-band diagram of NCNF/Ni-ZnO (15%) in (c) air and (d) H_2_S.

**Table 1:** Gas Sensing Behaviors of NCNF/Ni-ZnO Compared with Those in the Literature

sensing material	sensor response (%)	concentration (ppm)	response time (s)	working temperature (°C)	ref
NiO@ZnO nanotubes	47.5	50	50	215	^52^
Ni-ZnO nanorods	45.3	100	48	200	^53^
NiO/ZnO nanowire	31.5	100	15	RT	^54^
Fe_2_O_3_/NiO nanoplate	26.5	200	12	300	^55^
CuO-NiO core−shell microspheres	47.6	100	18	260	^56^
graphitic carbon-doped ZnO tubes	65.5	10	30	133	^57^
MoS_2_-GO	39.16	100	6.13	RT	^58^
ZTO-Ag@PPy	8	20	80	RT	^59^
CNTs/SnO2/CuO	2.6	100	240	RT	^60^
rGO/ZnO nanofibers	0.6	8	420	RT	^61^
Ru-CuO@ZIF-71	18.8	5	44	200	^62^
**NCNF/Ni-ZnO**	62.5	200	9.2	RT	**this work**

## 4 Conclusions

In conclusion, NCNF/Ni-ZnO nanocomposites with varying Ni-ZnO doping significantly improved gas-sensing performance for H_2_S detection. Among the different doping levels, NCNF/Ni-ZnO with 15% doping exhibited the maximum sensor response of 65.6% at 200 ppm with a response time of 9.2 s and a recovery time of 52.3 s, making it suitable for real-time detection. The electrospinning method ensured scalability and cost-effectiveness, further enhancing the potential for commercial applications. The excellent gas-sensing performance of NCNF/Ni-ZnO can be attributed to the synergistic effect of Ni-ZnO and the mesoporous NCNF structure, which provides a high surface area, abundant active sites, and enhanced gas adsorption and diffusion properties. This structural advantage also contributed to a faster response and recovery time, making the sensor highly efficient. The sensor showed remarkable stability, consistent responses over 30 days, and excellent resistance to humidity interference. The sensors showed excellent long-term stability (30 days) and effective humidity tolerance through a polynomial compensation approach that reduced RH-induced deviation by ∼4 times. Overall, this study highlights the potential of NCNF/Ni-ZnO nanocomposites as highly sensitive, stable, and efficient gas sensors with practical applications in environmental monitoring and industrial safety.

## Supplementary Material



## Notes

Humidity-compensation analysis, fitting equations, and cycling stability data (PDF)
