# Supplementary material for: Highly Sensitive and Selective Room-Temperature H2S Detection Using N-Doped Carbon Nanofiber/Ni−ZnO Composites
Source: ACS Appl Electron Mater. 2025 Dec 1;7(24):11027–37. doi: 10.1021/acsaelm.5c01903 (PMC12746459; doi:10.1021/acsaelm.5c01903)
Supplement: Supplementary file 1 [file el5c01903_si_001.pdf]

Supporting Information

Highly Sensitive and Selective Room-Temperature H_2S Detection Using N-Doped Carbon Nanofiber/Ni–ZnO Composites

Akshara Paras Parekh[a], Carlos Posada[a], Narayan Karmakar[b], Shilpa Jain*[c], Guoliang Liu[a,d,e*]

[a]Department of Chemistry, Virginia Tech, Blacksburg, VA 24061, USA

[b]Dwarkadas. J. Sanghvi College of Engineering, Mumbai-400056, India

[c]Department of Chemistry, Jai Hind College, Mumbai - 400020, India

[d]Macromoclecules Innovation Institute, Virginia Tech, Blacksburg, VA 24061, USA

[e]Division of Nanoscience, Academy of Integrated Science, Virginia Tech, Blacksburg, VA 24061, USA

Corresponding authors: gliu1@vt.edu; shilpa.jain@jaihindcollege.edu.in

1. Humidity Compensation Studies:

The sensor response shows strong dependence on relative humidity (RH). To describe this effect, the measured values were fitted using a second-order polynomial:

$R_{pred}(RH) = a\,(RH)^2 + b(RH) + c$………….. (S1)

Where R_{pred} denotes the predicted response at a given relative humidity (RH, %), a represents the quadratic coefficient, b is the linear coefficient, and c is a constant term. Least-squares fitting yielded:

$R_{pred}(RH) = -5.529 \times 10^{-4}\,(RH)^2 - 0.2311(RH) + 80.37$………...(S2)

(R^2 = 0.992, indicating an excellent fit to experimental data).

To remove RH-induced drift, the response at 11% RH (R_{base}= 77) was used as a reference. A correction factor (CF) was defined as:

CF(RH) = $\frac{R_{base}}{R_{pred}(RH)}$……………..(S3)

The humidity-compensated response (R_{corr}) was calculated as:

R_{corr}(RH) = R_{meas}(RH) x CF(RH)…………..(S4)

This normalization aligns responses at all RH levels to the 11% RH baseline, effectively reducing humidity-induced deviation and enabling more reliable gas sensing performance. To quantitatively correct for RH-induced drift, the measured response values at 11 %,32 %, 51 %, 63 % and 84 % RH were fitted to a second-order polynomial:

$R_{pred}(RH) = -5.529 \times 10^{-4}\,(RH)^2 - 0.2311(RH) + 80.37$………(S5)

(R^2 = 0.992)

Responses towards 200 ppm H2S were recorded for NCNF/ Ni-ZnO sensors (5, 10, 15 and 20 wt%) at RH = 11, 32, 51, 63, 84 %. For smooth, monotonic RH-dependence with minimal parameters (five RH points), we used a second-order polynomial fit for each composition, high orders did not materially change the trend and risk overfitting with this dataset.

Let $R_{means,i}(RH)$ be the measured response (%) for composition i at relative humidity RH (%). We fit

$\hat{R}_i(RH) = a_i RH^2 + b_i RH + c_i$ ……………….(S6)

Where a_i, b_i, c_i are least-squares coefficients (Table S1).

We define the baseline response at low humidity (11 % RH) from the experiment.

$R_{i,11} \equiv R_{means,i}(11\%)$……………….(S7)

A multiplicative humidity compensation factor aligns any RH to the 11 % baseline:

CF_i(RH) = $\frac{R_{i,11}}{\hat{R}_i(RH)}$, $R_{corr,i}$(RH) = $R_{meas,i}$(RH) x CF_i(RH)………(S8)

This preserves dose-response shape while removing RH bias.

To quantify humidity influence, we report two metrics over the RH set $\mathfrak{H} = \{11, 32, 51, 63, 84\}$:

Deviation (%) = $100 \times \frac{max_{\mathcal{H}} R - min_{\mathcal{H}} R}{max_{\mathcal{H}} R}$, …………(S9)

CV(%) = $100 \times \frac{SD\ (R)}{Mean\ (R)}$, ………..(S10)

Computed for both raw R_{meas} and corrected R_{corr}

Applying the polynomial RH compensation (Eq. S8) compresses the spread to CV ≈ 2.45–3.19% and deviation ≈ 6.1–7.3%. The 15 wt% Ni–ZnO sample shows the smallest residual drift after correction, matching its optimal catalytic/transport balance as shown in Figure S1.

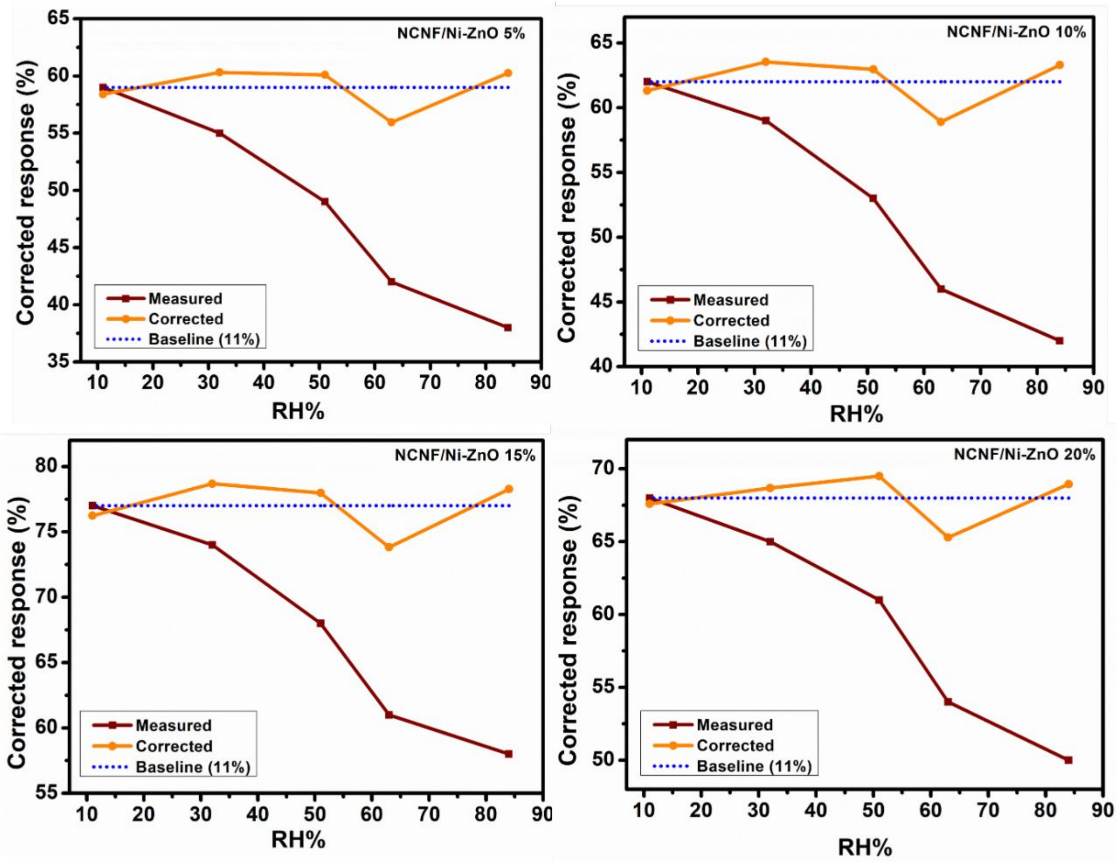

Figure S1. Measured (red) and RH-compensated (orange) responses of NCNF/Ni–ZnO sensors (5–20% Ni) at 200 ppm H_2S across varying RH. Compensation maintains responses within ±3% of the 11% RH baseline (blue).

Table S1. Polynomial coefficients (*a,b,c*) with baseline response at 11% RH, and deviation/CV values before and after compensation for NCNF/Ni–ZnO nanocomposites.

Composite	a	b	c	Baseline at 11% RH (%)	Deviation raw (max-min)/max (%)	Deviation corrected (%)	CV raw (%)	CV corrected (%)
NCNF/Ni-ZnO 5%	-0.0006	-0.2514	62.4402	59	35.5932	7.2377	17.9732	3.1899
NCNF/Ni-ZnO 10%	-0.001	-0.2015	65.0283	62	32.2581	7.2936	16.1144	3.1276
NCNF/Ni-ZnO 15%	-0.0006	-0.2311	80.3727	77	24.6753	6.1748	12.0451	2.6005
NCNF/Ni-ZnO 20%	-0.0013	-0.1368	70.0765	68	26.4706	6.0618	12.5895	2.454

Figure S1 demonstrates how polynomial fitting aligns the measured sensor responses of NCNF/Ni–ZnO composites at different RH levels to the 11% baseline, while Table S1 lists the corresponding coefficients and statistical metrics. The compensation reduces RH-induced variation by 4–6 times with the 15% Ni–ZnO nanocomposite showing the most stable and reproducible performance.

2. Humidity Cycling Stability Studies:

Humidity cycling tests were performed to evaluate sensor reproducibility under fluctuating moisture conditions, simulating real-world environments. The NCNF/Ni–ZnO nanocomposites were exposed to 200 ppm H_2S while RH was repeatedly cycled between low (11%) and high (84%) levels across three successive sequences. The measured responses were compared across cycles to assess retention of sensitivity and stability. Minimal deviation between cycles indicates that the nanocomposite effectively suppresses humidity-driven hysteresis by combining hydrophobic carbon backbones with oxygen vacancy rich Ni–ZnO interfaces, thereby ensuring stable operation under dynamic ambient conditions.

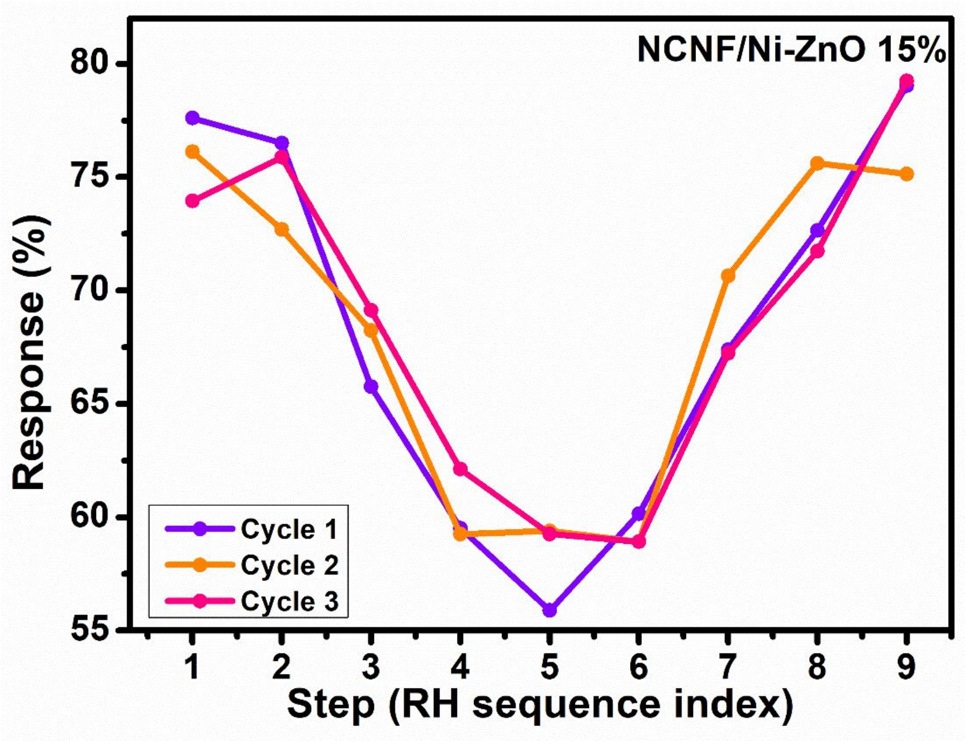

Figure S2. Repetitive RH cycling test for NCNF/Ni–ZnO (15 wt%) at 200 ppm H_2S showing stable response profiles across three consecutive humidity sequences (11–84% RH).
